# Common Behaviors and Faults When Doffing Personal Protective Equipment for Patients With Serious Communicable Diseases

**DOI:** 10.1093/cid/ciz614

**Published:** 2019-09-13

**Authors:** Joel M Mumma, Francis T Durso, Lisa M Casanova, Kimberly Erukunuakpor, Colleen S Kraft, Susan M Ray, Andi L Shane, Victoria L Walsh, Puja Y Shah, Craig Zimring, Jennifer DuBose, Jesse T Jacob

**Affiliations:** 1 School of Psychology, Georgia Institute of Technology, Atlanta; 2 School of Public Health, Atlanta Georgia State University; 3 Division of Infectious Diseases, Atlanta Department of Medicine; 4 Department of Pathology and Laboratory Medicine, Atlanta Emory University School of Medicine; 5 Division of Infectious Diseases, Department of Pediatrics, Emory University School of Medicine and Children’s Healthcare of Atlanta, Atlanta; 6 School of Architecture, Georgia Institute of Technology, Atlanta

**Keywords:** serious communicable disease, personal protective equipment, occupational health, human factors, risk analysis

## Abstract

**Background:**

The safe removal of personal protective equipment (PPE) can limit transmission of serious communicable diseases, but this process poses challenges to healthcare workers (HCWs).

**Methods:**

We observed 41 HCWs across 4 Ebola treatment centers in Georgia doffing PPE for simulated patients with serious communicable diseases. Using human factors methodologies, we obtained the details, sequences, and durations of doffing steps; identified the ways each step can fail (failure modes [FMs]); quantified the riskiness of FMs; and characterized the workload of doffing steps.

**Results:**

Eight doffing steps were common to all hospitals—removal of boot covers, gloves (outer and inner pairs), the outermost garment, the powered air purifying respirator (PAPR) hood, and the PAPR helmet assembly; repeated hand hygiene (eg, with hand sanitizer); and a final handwashing with soap and water. Across hospitals, we identified 256 FMs during the common doffing steps, 61 of which comprised 19 common FMs. Most of these common FMs were above average in their riskiness at each hospital. At all hospitals, hand hygiene, removal of the outermost garment, and removal of boot covers were above average in their overall riskiness. Measurements of workload revealed that doffing steps were often mentally demanding, and this facet of workload correlated most strongly with the effortfulness of a doffing step.

**Conclusions:**

We systematically identified common points of concern in protocols for doffing high-level PPE. Addressing FMs related to hand hygiene and the removal of the outermost garment, boot covers, and PAPR hood could improve HCW safety when doffing high-level PPE.

We identified ways that doffing protocols for high-level personal protective equipment may fail to protect healthcare workers. Hand hygiene, removing the outermost garment, boot covers, and respirator hood harbored the greatest risk and failed in similar ways across different hospitals.

## Teaser

Healthcare workers at 4 Ebola treatment centers were observed doffing personal protective equipment for simulated patients with serious communicable diseases. Using human factors methodologies, we identified common points of concern and conclude with potential interventions to alleviate these issues.

Prompted by the 2013–2016 outbreak of Ebola virus disease and its consequences in the United States, the Centers for Disease Control and Prevention (CDC) intensified efforts to deliver safer care for patients with suspected or known serious communicable diseases [1–3]. These diseases not only threaten the health of patients but potentially that of the healthcare workers (HCWs) responsible for their care. Personal protective equipment (PPE) and protocols for safe and efficient donning and doffing are important for protecting HCWs and preventing disease transmission [4], but risky behaviors during doffing do occur [5] and may result in self-contamination [6, 7]. Moreover, local adaptations of national guidelines result in variability in the PPE used and the procedures for donning and doffing PPE [2, 8, 9]. In the present study, we applied human factors methodologies to identify common obstacles to safe and efficient doffing across multiple Ebola treatment centers. We did this by first considering each hospital individually and then by inducing generalizations about most, if not all, hospitals.

## METHODS

We performed 41 simulations across the 4 state-designated Ebola treatment centers in Georgia. During each simulation, a single HCW donned high-level PPE for serious communicable diseases in his or her biocontainment unit, performed a standardized task (changing a urinary catheter bag on a mannequin), and then doffed according to his or her institutional protocol. There were 10 simulations at each site (11 at site A) with a different individual performing the role of the HCW each time. Most HCWs were nurses (90%), with the remainder comprising paramedics (5%) and HCWs with other roles (5%). All simulations involved a trained observer (TO); at 2 sites, the same individual served as TO for all or nearly all (90%) of the simulations. Six of the 10 simulations at site B occurred in a high-fidelity mockup of their biocontainment unit.

We recorded each simulation using 1 handheld and between 2 and 5 stationary cameras. Using these recordings (and the details of each site’s doffing protocol), we determined the duration of doffing steps, the different ways each step can fail to accomplish its goal(s) (ie, failure modes [FMs] [7]), and the frequency of each FM. FMs were determined via an Failure Modes and Effects Analysis, which is a technique for identifying and quantifying the risk of failures in a process in order to prioritize interventions that mitigate their effects. For each site, at least 2 human factors experts reviewed each simulation to identify FMs in the major doffing steps; judges considered elements such as knowledge of a site’s doffing protocol, the PPE likely to be contaminated, as well as evident human factors missteps (eg, errors of execution). Two human factors experts independently identified similar FMs that occurred at either most or all of the sites (ie, common FMs); reliability was assessed with percentage agreement.

For each site, 2 raters independently tallied the frequency at which each FM occurred in the simulations using the Observer XT version 12.5 (Noldus Information Technology, Leesburg, VA). Raw frequencies were transformed into a 5-point frequency scale. At each site, human factors and subject matter experts (eg, infectious disease physicians and nurses experienced in donning and doffing) independently rated the severity of the effect(s) of each FM using a 5-point scale that ranged from 1 (negligible) to 5 (catastrophic) [7]. For each FM at a site, we then calculated a risk index by multiplying the average severity rating for that FM by its transformed frequency value. We assessed the reliability of coding the frequency and sequence of FMs with Cohen’s kappa [10] and the reliability of the average severity rating of FMs with an average measures intraclass correlation [11].

Immediately after doffing, half of the HCWs at each site (n = 5) completed the NASA Task Load Index (NASA-TLX) [12] for the major doffing steps at their facility. The NASA-TLX comprises 6 subscales of workload (mental demand, physical demand, temporal demand, performance, effort, and frustration) that are each rated on a 100-point scale, with larger values corresponding to greater amounts of perceived workload.

## RESULTS

All doffing protocols involved removing boot covers, gloves (outer and inner pairs), the outermost garment (coveralls or a surgical gown), the powered air purifying respirator (PAPR) hood, the PAPR helmet/battery/belt, as well as repeated hand hygiene (with alcohol-based hand rub [ABHR] or disinfecting wipes) and handwashing with soap and water. These 8 common doffing steps are the focus of our analyses.

Across the sites, there were commonalities and notable variations in the order of doffing steps and the areas where steps occurred, the items of PPE used and how those items were removed, how hand hygiene was performed after PPE items were removed, and the role of the TO. The order of doffing steps (Table 1) followed a prototypical sequence of removing boot covers before outer gloves (except site C), followed by removing the outermost garment before the PAPR hood (except site C), which was followed by removing inner gloves before the PAPR helmet/battery/belt. At all sites, HCWs performed hand hygiene after each doffing step but only washed their hands with soap and water either immediately before or after removal of the PAPR helmet/battery/belt. At 3 sites, doffing began inside the patient room and finished in an anteroom. Doffing at site C, however, began in a clean room between the patient room and anteroom, then finished in the anteroom. Only at site A did HCWs remove the PAPR hood in the anteroom.

**Table 1.** Order of Doffing Steps at Each Site

	Sequence of Steps								
Site	1	2	3	4	5	6	7	8	9
A	Engage TO	Apron	Boot covers	Outer gloves	Tape	Outermost garment	PAPR hood	Inner gloves	PAPR assembly^a^
B	Engage TO	Apron	Boot covers	Outer gloves	Tape	Outermost garment	PAPR hood	Inner gloves	PAPR assembly^a^
C	Engage TO	Outer gloves	PAPR hood	Outermost garment	Boot covers	Inner gloves	Shoes^a^	PAPR assembly	Not applicable
D	Engage TO	Boot covers	Outer gloves	Tape	Outermost garment	PAPR hood	Inner gloves^a^	PAPR assembly	Headset

Abbreviations: PAPR, powered air purifying respirator; PAPR assembly, PAPR helmet/belt/battery; TO, trained observer.

^a^Step followed by handwashing with soap and water.

At all sites, HCWs wore washable shoes, disposable scrubs (except site C), and different colored pairs of gloves, with the outer pair having extended cuffs (Table 2). Only site C used surgical gowns rather than coveralls and gave HCWs the option to sit on a chair to remove their boot covers, rather than using a physical aid that required HCWs to stand. Site D was the only site where HCWs used their outer gloves (rather than inner gloves) to disconnect (ie, unsnap) the PAPR hood from the PAPR helmet. At sites A and D, HCWs removed their gloves using the “beaking method” (ie, creating a “beak” by pulling the inside surface of a glove over all 5 fingers); sites B and C used the glove-in-glove method [13].

**Table 2.** Items of Personal Protective Equipment Used at Each Site

Site	PAPR Hood	PAPR Assembly	Outermost Garment	Outer Gloves	Inner Gloves	Tape	Apron	Boot Covers	Scrubs	Shoes
A	Y	Y	Coveralls	Y	Y	Y	Y	Y	Y	Y
B	Y	Y	Coveralls	Y	Y	Y	Y	Y	Y	Y
C	Y	Y	Surgical gown	Y	Y	N	N	Y	Y	Y
D	Y	Y	Coveralls	Y	Y	Y	N	Y	Y	Y

Abbreviations: N, no; PAPR, powered air purifying respirator; PAPR assembly, PAPR helmet/belt/battery; Y, yes.

For hand hygiene, sites typically used ABHR; however, site C used disinfecting wipes (except in the final steps, when ABHR was used for bare hands). Site D was the only site to use automated ABHR dispensers exclusively and to enforce the duration of hand hygiene by having the HCW and the TO sing “Happy Birthday” aloud during each hand hygiene instance and 6 times during handwashing.

The role of the TO typically involved observing and verbally guiding the HCW with a written checklist. However, site C divided the observation of the HCW and reading the checklist between 2 observers. At site D, the (fully donned) TO helped remove the HCW’s PPE (eg, coveralls and PAPR hood).

### Duration of Doffing Steps

The median duration of complete doffing varied between sites, ranging from 6.9 to 22.2 minutes. The median duration of hand hygiene using ABHR between doffing steps (sites A, B, and D) was 16.3 seconds (interquartile range [IQR], 7.3–23.8). However, site D (where duration was enforced by singing a song) had a substantially larger percentage of hand hygiene instances that met the World Health Organization’s (WHO’s) recommendation [14] of 20 seconds or longer (75%) compared with site A (16%) and site B (11%). Site C used disinfecting wipes for hand hygiene predominantly (median duration, 25.5 seconds; IQR, 17.5–32.2).

**Figure 1.**
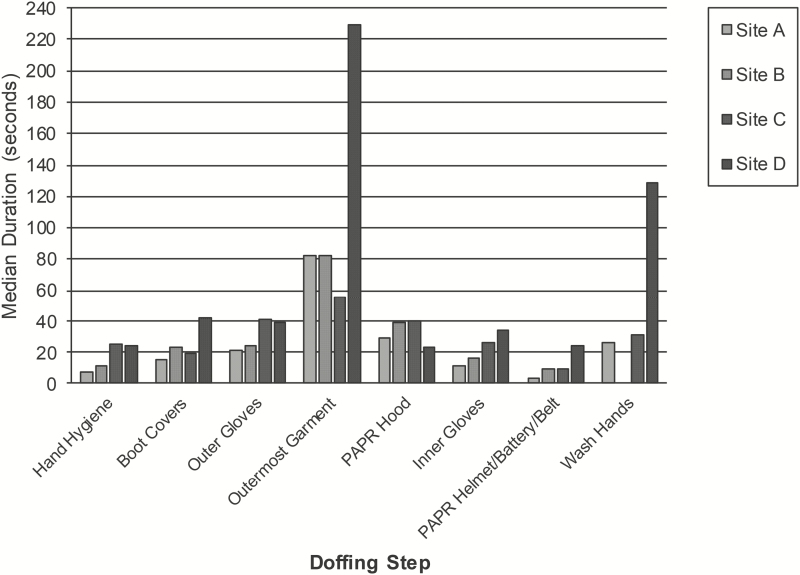
Median duration of the common doffing steps at each site. Note. “Wash hands” refers to cleaning hands with soap and water. At site B, handwashing was done as part of showering and was not observed. At site D, the trained observer removed the healthcare worker’s outermost garment. Abbreviation: PAPR, powered air purifying respirator.

Excluding hand hygiene and handwashing, the median duration of the common doffing steps was 25.2 seconds (IQR, 18.5–40.2; Figure 1). Removal of the outermost garment took the longest (median, 83.4 seconds; IQR, 55.4–116.5), especially at site D (median, 229.1 seconds; IQR, 181.2–268.7) where the TO helped remove the HCW’s coveralls. Outer glove removal (median, 34.7 seconds; IQR, 21.5–44.4) took longer than inner glove removal (median, 23.3 seconds; IQR, 13.5–34.4), except at site D. There was no appreciable difference in the duration of glove removal using the beaking method (median, 29.5 seconds; IQR, 17.5–44.1) and the glove-in-glove method (median, 28 seconds; IQR, 19.6–40).

### Failure Modes

The total number of FMs across all doffing steps varied between sites, ranging from 51 to 92. Regarding the number of FMs of the common doffing steps, hand hygiene and outermost garment removal were always in the top 3 at every site along with PAPR hood removal at all but 1 (site D), whereas PAPR helmet/battery/belt removal was always in the bottom 2 at every site.

**Figure 2.**
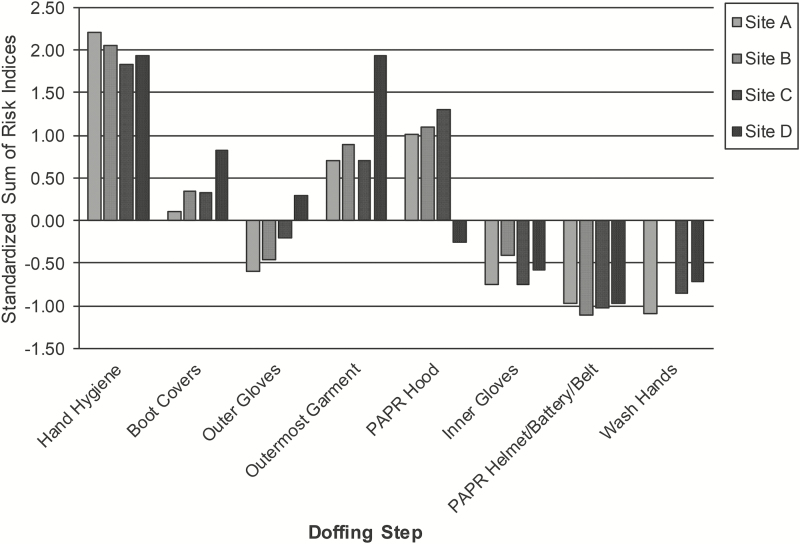
Standardized sums of risk indices of the common doffing steps at each site. Values are standard deviations above (positive values) or below (negative values) the mean sum of risk indices of individual doffing steps at each site. At site B, handwashing was done as part of showering and was not observed. At site D, the trained observer removed the healthcare worker’s outermost garment. Note: “Wash hands” refers to cleaning hands with soap and water. Abbreviation: PAPR, powered air purifying respirator.

The overall riskiness of a doffing step is indicated by the sum of the risk indices (ΣRIs) of the FMs associated with that doffing step [7]. Across sites, the mean reliability of coding the frequency and sequence of behaviors during the simulations was 0.71 (range, 0.61–0.79), corresponding to substantial agreement [15], and the mean reliability of the average severity ratings of FMs was 0.56 (range, 0.43–0.76), ranging from fair to excellent [16]. At all sites, hand hygiene and removal of the outermost garment and boot covers had above-average ΣRIs (Figure 2); at most sites (except site D), this was also true for PAPR hood removal. Notably, hand hygiene had a ΣRIs nearly 2 standard deviations (SDs) above each site’s mean ΣRIs. Removal of inner gloves and the PAPR helmet/battery/belt and handwashing had below-average ΣRIs at all sites; at most sites (except site D), the removal of outer gloves was also below average.

Summing across the common doffing steps at each site yielded 256 FMs (85% of the total number of FMs across all steps at all sites). Of these 256 FMs, 61 (24%) were judged to be occurrences of 1 of 19 common FMs identified at either most or all of the sites. Agreement on these common FMs was 84%, with differences resolved through discussion. Table 3 presents the RIs of the 19 common FMs, expressed in standard deviation units within each site to permit comparison between sites. Because the majority of the common FMs had RIs that were above average at each site, common FMs also tended to be risky FMs. Moreover, most sites had a common FM of glove removal with a RI that was >1 SD above their mean RI and at least 2 common FMs of hand hygiene with RIs >1.5 SDs (eg, failure to disinfect between fingers). Eliminating the common FMs of hand hygiene could reduce the overall riskiness of hand hygiene at each site by an average of 47%.

**Table 3.** Standardized Risk Indices of the Common Failure Modes

Common Doffing Step	Common Failure Mode	Site A	Site B	Site C	Site D
	Does not disinfect wrist.	2.08	1.20	N/A	1.21
	Does not rub gloves until dry.	1.90	0.87	0.46	−0.66
	Does not disinfect thumb.	1.48	1.53	2.58	1.21
Hand hygiene	Does not disinfect between fingers.	1.73	1.53	2.35	−0.04
	Gloves touch outside of PAPR hood shroud.	N/A	1.53	0.55	−0.58
	Shakes hands to dry.	0.78	−1.13	N/A	0.09
	Touches boot covers excessively.	−0.14	−0.08	−1.29	0.59
Boot cover removal	Crosses leg in front of self.	0.67	N/A	0.60	−0.66
	Whips boot cover off.	N/A	0.71	0.19	2.26
	Snaps glove.	−0.03	1.36	N/A	−0.41
	Whips glove off when removing.	N/A	−0.54	1.66	1.09
Glove removal	Difficulty pinching glove.	0.67	−0.15	−0.23	N/A
	Inner gloves touch outside of outer gloves.	N/A	−0.34	−0.46	0.34
	Coveralls are not contained on disinfectant mat.	−0.14	0.45	N/A	0.42
Outermost garment removal	Lower back is exposed.	−0.24	1.04	N/A	0.09
	Difficulty unsnapping PAPR hood from PAPR helmet.	N/A	−1.33	−0.23	0.09
	Touches PAPR hood excessively.	−1.01	0.18	−0.09	N/A
PAPR hood removal	Removes PAPR hood by pulling from front, not from back.	−0.14	1.23	0.19	N/A
	Inner gloves touch face shield.	0.65	1.69	−1.42	N/A

Values are standard deviations above (positive values) or below (negative values) the mean risk index at each site.

Abbreviations: PAPR, powered air purifying respirator; N/A, not applicable.

### Workload

The overall workload of the common doffing steps at each site was low (mean, 28.4; standard error [SE], 1.5), with no step at any site rated as extremely effortful or mentally, physically, or temporally demanding. The majority of the common doffing steps were rated as more mentally demanding (mean, 34.5; SE, 3.1) than physically (mean, 25.1; SE, 2.4) or temporally demanding (mean, 15.5; SE, 0.9), except at site D. The effortfulness of doffing steps was more strongly correlated with their mental demand (*r*, 0.76) than their physical (*r*, 0.64) or temporal demand (*r*, 0.55). However, this occasionally varied by doffing step. For example, the effortful steps of removing outer gloves had greater mental (mean, 47.8; SE, 6.5) than physical demand (mean, 29; SE, 4.7), but removing boot covers and the outermost garment had greater physical (mean, 50.7; SE, 5 and mean, 40; SE, 4.8, respectively) than mental demand (mean, 32.7; SE, 6.3 and mean, 36; SE, 5.8, respectively), except at site C. Although temporal demand was almost never the highest subscale for any step, it was the subscale most strongly correlated with frustration (*r*, 0.71). Finally, some steps were almost always greater in workload (eg, outer glove removal) than others (eg, inner glove removal), regardless of site and workload subscale.

## DISCUSSION

Despite variability in the high-level PPE used by hospitals and the details of their doffing protocols, we identified opportunities across hospitals for improving the safety of HCWs who care for patients with serious communicable diseases. 

Hand hygiene, considered the cornerstone of infection prevention, was the riskiest of the common doffing steps (except at site D). Of the 3 sites that primarily used ABHR for hand hygiene (sites A, B, and D), only site D enforced the duration of hand hygiene by singing “Happy Birthday” aloud and had a substantially larger percentage of hand hygiene instances that met WHO duration standards. Thus, the duration of hand hygiene may be improved through standardization, such as using an auditory tool (eg, singing a song or using a timer). Other opportunities for improving hand hygiene at most, if not all, sites were also apparent. No site ensured that all surfaces of the HCW’s hands were covered, nor were their hands consistently rubbed until dry. There were also instances of incidental contact with potentially contaminated PPE, such as the outside of the PAPR hood shroud, during hand hygiene, (Table 3). HCWs at most sites, with the exception of the one site that predominantly used disinfecting wipes for hand hygiene instead of ABHR (site C), occasionally shook their hands dry and did not consistently disinfect their wrists. Despite the redundancy of having multiple hand hygiene steps in doffing protocols, the task of hand hygiene warrants close attention in the development and execution of doffing protocols. Moreover, the ubiquity of these FMs in biocontainment units may also warrant further assessment of hand hygiene in routine patient care using similar multidisciplinary approaches.

Removal of the outermost garment (coveralls or surgical gown) was always among the riskiest doffing steps, requiring the most time and being rated as one of the most effortful steps. The primary task demands tended to be physical rather than mental or temporal, particularly for coveralls. For sites that used coveralls, the HCW’s lower back occasionally became exposed during removal, but this was not observed at site C, where HCWs wore surgical gowns. During removal, coveralls were also difficult to keep within the confines of the disinfectant mat. These FMs were not eliminated by having the TO remove the HCW’s coveralls (site D), which was also associated with the longest doffing duration of all the sites. The decision to use a surgical gown or coverall as the outermost garment should consider these findings, as well as HCW comfort and material durability.

Outer glove removal was effortful (except at site B), mentally demanding, and, compared with inner glove removal, took longer to perform (except at site D) and was greater in workload. Each site had a common high-risk FM associated with their glove removal protocol, such as whipping gloves (ie, pulling a glove off abruptly, not smoothly) or snapping gloves (ie, a glove recoils sharply after losing one’s grasp). However, the method of glove removal (beaking vs glove-in-glove) did not appear to impact the duration of doffing, workload, or pattern of FMs. These FMs may instead be related to other factors, such as the physical characteristics of gloves (eg, stiffness [17, 18]). Because gowns and gloves are frequently used as part of contact isolation precautions, these findings may also have implications for routine patient care.

PAPR hood removal was risky except at site D. Self-removal of the PAPR hood led HCWs to touch the outside of the hood excessively with their inner gloves, remove the hood by pulling from the front (rather than from the back), and touch the face shield with their inner gloves. Having the TO remove the PAPR hood (site D) appeared to mitigate excessive touching and improper removal. However, the decision to have the TO vs HCW remove the PAPR hood should consider the risk(s) to both the TO and HCW.

At each site, removal of boot covers was a moderately risky doffing step that was the most physically demanding (except at site C, where HCWs could sit or stand), with each site seeing HCWs touch their boot covers excessively as well as instances of other common FMs, such as whipping off boot covers. Design strategies may ameliorate the physical demands of this (and other doffing steps) by using the physical environment to promote easier doffing (eg, via effective stabilization aids [19, 20]).

Additionally, some common problems may also benefit from similar interventions not observed in the present study. Having the TO provide greater supervision during hand hygiene, such as asking, “Are your hands dry?” at the end of each hand hygiene instance, could address common high-risk components of doffing. Other interventions that target the immediate environment, such as installing a mirror for the HCW, may mitigate touching PPE (eg, the PAPR hood or boot covers) inappropriately [17, 18]. Finally, some common weaknesses in doffing protocols may particularly benefit from training (eg, the thoroughness of hand hygiene [21]).

Because doffing protocols are complex, and our observations may also reflect variables that we did not assess directly (eg, the culture of the team or the quality of HCW training), we cannot extrapolate a single “correct” approach to the entire process nor even for an individual step(s). Since any change may lead to unintended consequences downstream in the doffing process, potential modifications spurred by our findings should consider the doffing process carefully and holistically.

The common points of concern that we identified, particularly those that have also emerged in related research efforts (eg, not rubbing hands thoroughly during hand hygiene [22]), underscore the need for a systems engineering approach to addressing such problems in healthcare [22, 23]. To this end, leveraging the expertise of human factors engineers and healthcare practitioners can help organizations identify, understand, and remediate problems that may otherwise go unnoticed [8, 22], providing solutions not only for biocontainment units but also for more routine clinical settings.

## Notes

***Acknowledgments.*** The authors thank Eric Stearman and Jeremy Posey for help coding and analyzing data. The authors also thank the participating healthcare workers, hospitals, and team members of the Prevention Epicenter of Emory and Atlanta Consortium Hospitals.

***Financial support.*** This work was supported by the Prevention Epicenters Program of the Centers for Disease Control and Prevention (award U54CK000164). Emory University’s participation was supported in part by the National Center for Advancing Translational Sciences of the National Institutes of Health (award UL1TR000454; Atlanta Clinical and Translational Science Institute).

***Supplement sponsorship. This article appears as part of the supplement “Personal Protective Equipment for Preventing Contact Transmission of Pathogens: Innovations from CDC’s Prevention Epicenters Program,” sponsored by the CDC’s Prevention Epicenters Program.***

***Potential conflicts of interest.*** All authors report no potential conflicts. All authors have submitted the ICMJE Form for Disclosure of Potential Conflicts of Interest. Conflicts that the editors consider relevant to the content of the manuscript have been disclosed.
